# Evaluating the Impact of Mask Mandates and Political Party Affiliation on Mental Health Internet Search Behavior in the United States During the COVID-19 Pandemic: Generalized Additive Mixed Model Framework

**DOI:** 10.2196/40308

**Published:** 2023-03-03

**Authors:** Joseph A Gyorda, Damien Lekkas, George Price, Nicholas C Jacobson

**Affiliations:** 1 Center for Technology and Behavioral Health Geisel School of Medicine Dartmouth College Lebanon, NH United States; 2 Quantitative Biomedical Sciences Program Dartmouth College Hanover, NH United States; 3 Department of Biomedical Data Science Geisel School of Medicine Dartmouth College Hanover, NH United States; 4 Department of Psychiatry Geisel School of Medicine Dartmouth College Hanover, NH United States

**Keywords:** mental health, Google Trends, mask mandates, political party, generalized additive mixed modeling, COVID-19

## Abstract

**Background:**

The impacts of the COVID-19 pandemic on mental health worldwide and in the United States have been well documented. However, there is limited research examining the long-term effects of the pandemic on mental health, particularly in relation to pervasive policies such as statewide mask mandates and political party affiliation.

**Objective:**

The goal of this study was to examine whether statewide mask mandates and political party affiliations yielded differential changes in mental health symptoms across the United States by leveraging state-specific internet search query data.

**Methods:**

This study leveraged Google search queries from March 24, 2020, to March 29, 2021, in each of the 50 states in the United States. Of the 50 states, 39 implemented statewide mask mandates—with 16 of these states being Republican—to combat the spread of COVID-19. This study investigated whether mask mandates were associated differentially with mental health in states with and without mandates by exploring variations in mental health search queries across the United States. In addition, political party affiliation was examined as a potential covariate to determine whether mask mandates had differential associations with mental health in Republican and Democratic states. Generalized additive mixed models were implemented to model associations among mask mandates, political party affiliation, and mental health search volume for up to 7 months following the implementation of a mask mandate.

**Results:**

The results of generalized additive mixed models revealed that search volume for “restless” significantly increased following a mask mandate across all states, whereas the search volume for “irritable” and “anxiety” increased and decreased, respectively, following a mandate for Republican states in comparison with Democratic states. Most mental health search terms did not exhibit significant changes in search volume in relation to mask mandate implementation.

**Conclusions:**

These findings suggest that mask mandates were associated nonlinearly with significant changes in mental health search behavior, with the most notable associations occurring in anxiety-related search terms. Therefore, policy makers should consider monitoring and providing additional support for these mental health symptoms following the implementation of public health–related mandates such as mask mandates. Nevertheless, these results do not provide evidence for an overwhelming impact of mask mandates on population-level mental health in the United States.

## Introduction

### Background

For >2 years, the COVID-19 pandemic has been afflicting global populations in a variety of ways. Although the physical impact of COVID-19 is evident and has been well documented in the literature [1-3], more research is needed to assess the mental impact of the COVID-19 pandemic. Many studies have assessed short-term impacts of the pandemic on mental health. For instance, a meta-analysis by Talevi et al [4] revealed impacts on psychological well-being, including increased anxiety, depression, stress, and insomnia during the first few weeks of the pandemic in March 2020 and April 2020. These associations were detected in a variety of populations, including college students, health care workers, and patients with COVID-19 [4]. Other works have unveiled similar mental impacts because of extended periods of quarantine [5,6]. In light of these findings, fewer works to date have studied the long-term impacts of the COVID-19 pandemic on mental health. In a meta-analysis, Bourmistrova et al [7] found that patients with COVID-19 had mild to no anxiety and depression symptoms for at least 1 month following diagnosis, but these trends were not investigated in the general population (eg, including patients without COVID-19). Conversely, Veldhuis et al [8] found that survey participants (N=1567) had increased depressive symptoms and suicidal thoughts after a 5-month follow-up at the end of summer 2020. Taken together, more research is still needed to assess the long-term mental effects of COVID-19, the factors contributing to these effects, and the populations most at risk of experiencing these effects. Such research will help accentuate the need to institute policy measures that increase access to and strengthen mental health services worldwide as well as increase awareness of the specific mental risk factors that exist among high-risk populations [5]. In this light, this study aimed to contribute to the limited literature on the long-term mental health impacts of COVID-19 through investigating trends in mental health activity along with their possible contributing factors over the first year of the pandemic (March 2020-March 2021) in a large representative national sample in the United States.

Importantly, a data stream that has shown promise for operationalizing mental health is internet search term activity [9,10], with dynamic tools such as Google Trends providing a publicly available platform with which to investigate internet search behavior. Most people aged <25 years perform internet searches as their primary means of seeking mental health help [11]; therefore, internet search activity may be a potent indicator of changes in mental health. Various studies have documented the use of internet search engines for mental health self-diagnosis [9,12]. In addition, myriad works have connected internet searches for psychopathologies with actual experience of them. For instance, a study found that increased self-reported anxiety was associated with increased Google search rates for anxiety [13], whereas another study unveiled a positive relationship between depressive symptom search volume and same-month suicide rates [14]. In the context of COVID-19, Ayers et al [15] discovered that there were sudden spikes in anxiety-related search activity in March 2020 to April 2020 in the United States. Furthermore, analyses by Gianfredi et al [16] revealed significant positive associations between mental health–related search volume (eg, insomnia, suicide, and depression) and COVID-19 deaths. Although it is important to acknowledge that mental health symptoms differ greatly across individuals, analyzing trends in population-level internet search behavior can reveal associations with potentially important implications for public health and policy [14].

Leveraging internet search behavior as an indicator of mental health, this work aimed to specifically disentangle some of the factors that may influence changes in mental health in relation to the COVID-19 pandemic. In particular, this research brought to attention the ubiquity of state-enforced mask mandates throughout the pandemic and their potential to affect mental health. A total of 39 out of 50 states in the United States implemented statewide mask-wearing mandates as a means of limiting the transmission of the novel coronavirus. Although mask mandates may have provided some with a reduction in anxiety because of added protection from the coronavirus or mitigating sources of social anxiety [17], wearing a face mask may be perceived as restrictive or uncomfortable [18] as well as something that may threaten personal freedom [19]; therefore, it is reasonable to believe that mental reactions to the requirement of masking were heterogeneous and often adverse both across the country and throughout this phase of the pandemic. Reasons for an adverse response are manifold and need not be restricted to groups rather than to masking. For example, individuals in favor of masking may respond negatively to the imposed changes as a result of interactions with those that are less in favor of masking. A study revealed that there was an increase in anger present in tweets that were promasking in states both with and without mask mandates [19]. In light of the associations between mask mandates and mental health, no studies have examined the broader, long-term mental impact of mask mandates. In addition, there is evidence suggesting that masking behaviors, including mental responses to masking mandates as well as mental health constructs such as anxiety and fear, differ across political parties in relation to the COVID-19 pandemic [20,21]. Accordingly, this study sought to investigate the associations among state-level mask mandates, political affiliations, and mental health during the COVID-19 pandemic by way of internet search behavior. Such an investigation may provide leaders with important considerations regarding the long-term mental implications of implementing large-scale interventions such as mask mandates and how the political leanings of their populations may contribute to these mental health responses.

### Objectives

The aim of this study was to evaluate the long-term influences of mask mandate implementation and political party affiliation on mental health search term volume between March 24, 2020, and March 29, 2021, in the 50 states in the United States. Changes in mental health were modeled using generalized additive mixed models (GAMMs) on data collected through Google Trends. In particular, this study serves to complement the work by Jacobson et al [22], where an attenuation of mental health search term activity was found in the United States after stay-at-home orders were implemented. Consequently, this work was interested in examining whether statewide mask mandates and political party affiliation, quantified as political party elected during the 2020 presidential election (see the *Mask Mandates and Political Party Elected* section), had similar significant associations with mental health activity. In addition, physical health symptom search terms both related and unrelated to COVID-19 were analyzed as a means of comparison with the associations between mental health search term volume and mask mandates. In this data-driven exploratory analysis, the authors investigated the research questions outlined in Textbox 1.

**Research questions and hypotheses.**

**Research question 1**

- Were statewide mask mandates significantly associated with the trajectories of internet mental health search queries in the United States between March 2020 and March 2021 compared with states without mask mandates in effect, and were these mandates differentially associated with the trajectories of mental health search queries as a function of state political party affiliation?

***Hypothesis 1***

- Given the evidence suggesting a mental impact of mask mandates [17-19] and evidence suggesting a differential mental impact of mask mandates across political party affiliation [20,21], we expect to uncover significant associations (either positive or negative) between statewide mask mandates and the trajectories of mental health search queries, along with differential associations across political parties.

**Research question 2**

- Would the associations between statewide mask mandates and political party elected be unique to the trajectories of internet mental health search queries or would they be consistent with the trajectories of internet physical health queries both related and unrelated to COVID-19?

***Hypothesis 2***

- In line with the findings of Jacobson et al [22], we expect the associations between statewide mask mandates and political party elected to be unique to the trajectories of mental health search queries.

## Methods

### Search Term Data Collection

Given the prominence of Google as a search engine (eg, Google accounts for roughly 88% of the search engine market in the United States [23]), these analyses leveraged Google Trends, which allows for public access to search term volume for a given time and location. Several studies have leveraged Google Trends data to examine how mental health information is sought out [22,24,25]. As can be found in the documentation for Google Trends, the raw counts for a given search term are normalized by location and time of search and then scaled to a number from 0 to 100 representing the proportion of searches on all topics that the given term constitutes. Such normalization allows for easier comparison across geographic regions, where population may play a significant role in relative search term popularity. This analysis downloaded data from Google Trends using the *gtrendsR* package in R (version 1.4.8; R Foundation for Statistical Computing) [26]. To obtain data with the most granularity, hourly trend data were queried. The areas of interest were the 50 states constituting the United States; thus, search terms were normalized across states. As Google Trends only stores hourly data for up to 7 days, hourly data were programmatically pulled each Monday from March 23, 2020, to March 29, 2021. This period of 372 days spans the early days of the pandemic to the widespread availability of the COVID-19 vaccine in the United States. During this time frame, 39 states implemented statewide mask mandates, most of which went into effect before or during the summer of 2020. Thus, the given time frame allowed for careful introspection into the short- and long-term effects of mask mandate implementation on mental health search term activity.

The following 19 mental health search terms were queried from Google Trends, as described previously: “anxiety,” “depression,” “ocd” (obsessive-compulsive disorder), “hopeless,” “angry,” “afraid,” “apathy,” “worthless,” “worried,” “restless,” “irritable,” “tense,” “scattered,” “tired,” “avoiding,” “procrastinate,” “insomnia,” “suicidal,” and “suicide.” Aligning with previous work by the authors [10,22], these terms were validated from previous research on using Google Trends to assess mental health [27], as well as from previous research assessing rapid affective symptom changes as defined by the Diagnostic and Statistical Manual of Mental Disorders, Fifth Edition [28,29]. In addition to these terms, physical health search terms, both without known associations to COVID-19 (“abrasion,” “allergic,” “angina,” “apnea,” “bleeding,” “blister,” “bruising,” “conjunctivitis,” “constipation,” “discharge,” “earache,” “flatulence,” “fracture,” “hemorrhage,” “incontinence,” “inflammation,” “itching,” “lesions,” “rash,” “spasms,” “swelling,” and “syncope”; 22 terms) and with known associations to COVID-19 (“bloating,” “blurry,” “congestion,” “cough,” “coughing,” “croup,” “diarrhea,” “dizzy,” “fainting,” “fever,” “pain,” “sneezing,” “strep,” “stuffy,” and “vomiting”; 15 terms) were queried to ascertain whether any significantly detected patterns in mental health search term activity were unique to and distinct from those pertaining to physical health. Note that each mental and physical health search term was considered independently in this study; in other words, composite scores aggregating the individual search term counts to create a composite score capturing *total* mental and physical health activity were not created. This decision was made because combining individual search terms with differential trends throughout the pandemic may attenuate these individual trends in the composite score such that the composite score may not be reflective of changes in specific mental or physical health symptoms, therefore making it uninformative.

### Preprocessing

#### Outcome Calculation and Imputation

All mental and physical health search term data were queried using the *gtrendsR* package [26] and read into R (version 4.0.3). As this study was interested in investigating the long-term associations between mask mandates and search term activity, the raw, hourly state-specific search term counts collected through Google Trends were first aggregated into state-specific daily hit counts (eg, daily counts of search volume) to improve the interpretability of the models described in the *GAMM Approach* section. When performing hourly aggregation, 3 decisions were made. First, data from March 23, 2020—the first day in the time frame of interest—were dropped as the weekly programmatic collection of data initially started at approximately 7 PM EST. Accordingly, each state had <24 hours of data available for this date. Thus, the time frame of interest was shortened to 371 days, from March 24, 2020, to March 29, 2021. Second, some states had search term hit counts listed as “<1” for a given hour by Google Trends. These entries were coded as “0” to perform hourly aggregation. Third, throughout the data collection period, there were periods where certain states were missing hourly hit counts for a given search term. Some days were only missing a few hours for a given state and search term, whereas some days were entirely missing (eg, all 24 hours). Each search term had some degree of missingness (Tables S1 and S2 in Multimedia Appendix 1), which needed to be addressed before modeling could occur.

To address the issue of missingness in hourly search term hit counts, zero imputation was implemented, where missing hours were inserted into the data set and had hit counts coded as “0.” Instances of missing hourly hit counts implied that there was too low of a search prevalence for a term’s data to be recorded. As such, this method of imputation is an appropriate strategy to address missing hourly data. A summary of the relative number of missing hours and days for each search term can be found in Tables S1 and S2 in Multimedia Appendix 1. Only search terms with <5% missingness across the study’s data collection period were included to avoid drawing spurious conclusions in subsequent modeling. Once these search terms were identified, zero imputation was performed as described previously to ensure that each term had a continuous time window. Thus, the data leveraged for these analyses were data sets corresponding to each search term, each data set having 18,550 rows (371 days for each of the 50 states), with each row corresponding to an aggregated normalized hit count for a given day in a given state. These aggregated normalized hit counts were used as the outcomes of interest for the analyses.

#### Mask Mandates and Political Party Affiliation

Data pertaining to the implementation of statewide mask mandates were collected from the article by Hubbard [30]. Statewide mask mandates were considered to be masking mandates implemented with the intent of applying to all members of a state’s population (eg, not countywide mandates or mandates restricted to schools or specific businesses). Using the mask mandate data for each state, a binary variable was created with values corresponding to each day in the 371-day window—values were coded as “1” for a given state on a given day if the mask mandate was in effect and “0” if the mask mandate was not in effect. States that at no point had a statewide mask mandate were coded as “0” for each day. Along with mask mandate data, state political party affiliation was defined as the result of the popular vote in the 2020 presidential election, which was collected from CNN [31]. From this, another binary variable was defined: states received a “1” if the Republican presidential candidate won the popular vote and “0” if the Democratic candidate won. Note that the candidate winning the popular vote in each state also won the electoral votes for that state, and in cases where states allowed electoral votes to be split (eg, Maine and Nebraska), the candidate winning the majority of the electoral votes also won the popular vote [31]. Therefore, state political party affiliation was quantified by the party of the candidate elected by each state for the 2020 election and is henceforth referred to as the “political party elected.” For reference, Table S1 in Multimedia Appendix 2 contains data corresponding to statewide mask mandates and political party elected. A third and final binary variable was defined by taking the product of the mask mandate and political party variables. In this manner, states received a “1” for a given day if their political party elected was Republican and a statewide mask mandate was in effect and a “0” otherwise. These 3 binary variables served as predictors with which to model the associations of interest, as described in the *GAMM Approach* section.

#### Model Covariates

These analyses also sought to take into account other socioeconomic factors that could influence mental health activity such that controlling for these variables in modeling would reduce the chance that any significant associations related to mask mandates and political party elected were owing to chance or random noise. To account for variations in the severity of the pandemic across states over time, variables pertaining to statewide COVID-19 cases and deaths were included in the model as there is empirical evidence associating increases in COVID-19 cases [32] and deaths [33] with fluctuations in mental health activity. State-level data for COVID-19 cases and deaths were collected from a GitHub repository curated by The New York Times [34], where values represent the cumulative case and death counts throughout the pandemic. These values were recoded to represent new daily cases and deaths to reflect the day-to-day severity of the pandemic more clearly.

Other factors influencing mental health outcomes are urbanization and income. Individuals living in highly populated urban areas have been shown to experience worsened mental health outcomes because of social disparities, pollution, lack of nature, and other aspects associated with urban living [35]. In addition, low-income households are at a higher risk of mental health problems and are simultaneously less able to access mental health services [36]. Therefore, both urbanization and income may have affected how individuals and communities responded mentally to the pandemic. Thus, this analysis chose to incorporate these 2 additional variables because of their potentially significant and marginal influence on the mental health of an ailing population. State-level data for urbanization were collected from the 2010 census [37], where values for each state reflect the percentage of the population estimated to be living in an urban area. State-level data for per capita income were collected from a 2020 data set published by the US Department of Commerce [38], where values for each state represent the average per capita income in thousands of dollars.

### GAMM Approach

This study leveraged GAMMs to examine changes in mental health search trends over the first year of the COVID-19 pandemic. GAMMs are a powerful modeling approach as they can account for nonlinear trends as well as disentangle the interdependence of observations. This analysis used data in which observations (ie, daily search term counts) were nested within groups (ie, states); therefore, incorporation of a mixed-effects framework was suitable to estimate trajectories of search term activity over time. In addition, GAMMs allow for estimations of variable-specific trend comparisons (smooths) via incorporation of smoothing functions (smoothers) [39]. Specifically, this study used splines as the smoothing function as splines account for nonlinearity in the data but only model nonlinearity in the predictor-outcome relationship if nonlinearity provides the best fit for the data [22]. To implement GAMMs, the *bam* function within the *mgcv* package was used. The *bam* function allows for the specification of GAMMs, is optimized for faster run times on larger data sets (eg, each search term had data for 371 days and 50 states, or 18,850 individual observations), and allows for direct hypothesis testing of the estimated smooths [40].

The selected model incorporates 8 smooth terms, denoted by “s,” as well as 6 linear terms pertaining to state-level mask mandate, political party elected, urbanization, per capita income, and COVID-19 cases and deaths. These linear terms were included to isolate the main effects of the binary terms of interest, allowing the smooth terms to capture any nonlinear relationships in the data. Importantly, the negative binomial distribution was chosen for the model because of the use of (nonnormally distributed) count data and overdispersion (ie, variance exceeds the mean) present in the outcome variables. Textbox 2 describes the complete model architecture with associated variable definitions. Each model was independently run on 12 mental health and 26 physical health search terms.

**Complete model architecture and variable definitions.**

**Model architecture**

- Outcome_*i,j*_ ~ s_1_(Time_*i,j*_) + s_2_,i(Time_*i,j*_) + s_3_(TimeSM_*i,j*_) × MaskMandate_*i,j*_ + s_4_(TimeSM_*i,j*_) × MandateParty_*i,j*_ + s_5_(TimeSM_*i,j*_) × MandateUrbanization_*i,j*_ + s_6_(TimeSM_*i,j*_) × MandateIncome_*i,j*_ + s_7_(TimeSM_*i,j*_) × MandateCases_*i,j*_ + s_8_(TimeSM_*i,j*_) × MandateDeaths_*i,j*_ + MaskMandate_*i,j*_ + MandateParty_*i,j*_ + MandateUrbanization_*i,j*_ + MandateIncome_*i,j*_ + MandateCases_*i,j*_ + MandateDeaths_*i,j*_

**Variable definitions**

- Outcome_*i,j*_: the total search volume of a mental health or physical health term for state *i* at time *j*
- Time_*i,j*_: the number of days elapsed from March 24, 2020, to time *j*
- TimeSinceMandate_*i,j*_ (abbreviated as TimeSM_*i,j*_): a time difference variable that measures, for a specific state *i* at time *j*, the time between *j* and the implementation of the statewide mask mandate policy for state *i*; this variable was defined as “0” when *j* and the date of policy implementation were equivalent and negative if *j* occurred before the date of policy implementation
- MaskMandate_*i,j*_: a dummy variable encoded as “0” for all *j* when the mask mandate was not in place for *i* and “1” for all *j* when the mandate was in place for *i*
- MandateParty_*i,j*_: a dummy interaction variable calculated as MaskMandate_*i,j*_ × PoliticalParty_*i*_ and, thus, encoded as “1” when the mask mandate was in place for a Republican state *i* at time *j* and “0” otherwise
- MandateUrbanization_*i,j*_: an interaction variable calculated as MaskMandate_*i,j*_ × Urbanization_*i*_ and, thus, encoded as the urbanization value of *i*—the percentage of the population living in an urban area—if the mandate was in effect at *j* and “0” otherwise
- MandateIncome_*i,j*_: an interaction variable calculated as MaskMandate_*i,j*_ × PerCapitaIncome_*i*_ and, thus, encoded as the average per capita income value in dollars for *i* if the mandate was in effect at *j* and “0” otherwise
- MandateCases_*i,j*_: an interaction variable calculated as MaskMandate_*i,j*_ × CovidCases_*i,j*_ and, thus, encoded as the number of new COVID-19 cases for state *i* at time *j* for Republican states with a mask mandate in effect and “0” otherwise
- MandateDeaths_*i,j*_: an interaction variable calculated as MaskMandate_*i,j*_ × CovidDeaths_*i,j*_ and, thus, encoded as the number of new COVID-19 deaths for state *i* at time *j* for Republican states with a mask mandate in effect and “0” otherwise

All *s* terms indicate that smoothing was used to estimate the relationship between the predictor and outcome of interest. Term s_2_ represents the random smooth slopes of state *j* at each time *i*, allowing a linear or nonlinear random effect to account for changes in the outcome. Terms s_3_ to s_8_ represent varying coefficient smooths specified by the tensor product interaction smoothing function, which, in addition, accounts for the presence of main effects in the interacting variables [40]. For model-specific hyperparameter specification, “fREML” (“fast REML” computation) was used for smoothing parameter estimation, which, when used in conjunction with setting *discrete*=TRUE, discretizes covariates into smaller bins and substantially reduces computation time [41]. Furthermore, the negative binomial distribution was used in the model by selecting *family*=“nb.”

In terms of smooth-level hyperparameter specification, all smooth terms, aside from s_2_, were modeled with a thin plate regression spline (TPRS) basis function, which allows for linear or nonlinear relationships between the predictor and the outcome variable while also penalizing nonlinearity such that the data will only be modeled as nonlinear if the model fit is substantially better. In addition, modeling with a TPRS circumvents issues with knot placement [42]. Term s_2_ uses a factor smooth interaction basis function that fully accounts for random effects; provides a better fit when the number of grouping variables is high [39], as in these analyses (ie, 50 states); and draws group-level smooth terms toward zero by only estimating 1 smoothing parameter across groups [41]. Along with this, the group-level smoothers, s_3_ to s_8_, use m=1, which penalizes the TPRS with the squared first derivative of the function, whereas s_1_ and s_2_ use m=2, penalizing the spline with the squared second derivative of the function. Using m=1 in the group-level smoothers reduces collinearity with the global smoother and group-specific terms (ie, s_1_ and s_2_) [41]. In addition, the smooth terms s_3_ to s_8_ use the default *k* value (*k=*5) in *mgcv*, which balances smooth fitting with computational time [40]. However, *k* is set to 3 for the smooth terms s_1_ and s_2_ to allow for up to cubic trends to be modeled by the time and state random effects of time variables. Although it is optimal to choose a high *k* when computationally feasible, limiting *k* in this context restricts the nonlinear deviations in the time trends that can be explained by the state random effects such that these complex state-specific nonlinearities can be potentially accounted for by other sources.

This study is interested in examining the associations among mask mandates, political party elected, and search term trajectory; therefore, emphasis is placed on the s_3_ and s_4_ terms. Term s_3_ estimates the observed associations of the statewide mask mandate as a deviation from the state-specific counterfactual trend that would have occurred had there not been a statewide mask mandate implemented, and s_4_ estimates the observed associations of Republican states with a statewide mask mandate as a deviation from the state-specific counterfactual trend that would have occurred in Democratic states with a statewide mask mandate. Accordingly, the model output from s_3_ and s_4_ is of particular interest.

### Model Assessment

These analyses consisted of 38 negative binomial GAMMs, with each model corresponding to the volume of activity for 1 search term over time as the outcome. The results of the smoothing term estimation were of particular interest to assess any nonlinear associations captured by the predictor variables. As smooth terms estimate nonlinear trends in the data, the results of these models are not equivalent to a regression slope; rather, estimated df (EDF) are reported, where EDF=1 corresponds to a linear relationship between the predictor and outcome, 1<EDF≤2 corresponds to a weak nonlinear relationship, and EDF>2 corresponds to a strong nonlinear relationship [43]. The *bam* function in *mgcv* performs a significance test and reports the *P* value for the EDF corresponding to each smooth term. Importantly, as this analysis consisted of 12 independent models for mental health search terms, results of variable significance needed correction for multiple comparisons. Accordingly, *P* values were adjusted by controlling the false discovery rate using the Benjamini-Hochberg procedure. The same procedure was used for the 17 independent not COVID-19–related and 9 independent COVID-19–related physical health search term models.

To assess how well the models explained the variation in daily search term counts, the *R*^2^ value was reported. This *R*^2^ value corresponds to the conditional *R*^2^, or the total variance in the outcome explained by both the fixed and random effects. Thus, to assess whether the random effects are driving model prediction as well as determine if the fixed effects (ie, mask mandate and political party elected) explain a considerable amount of the variation, the marginal *R*^2^—the total variance explained by the fixed effects—was also reported. Given limitations in the ability to directly estimate the marginal *R*^2^ from models constructed using the *mgcv* package, the marginal *R*^2^ was estimated manually by rerunning each model without the s_2_ random effect of time and reporting its *R*^2^ value.

Note that, as MaskMandate*_i,j_* was coded as a “1” when a state had a mask mandate in effect and “0” when the mandate was not in effect or the state did not have a mandate, these analyses only make interpretations of the associations between mask mandates and search term trajectory *after* the implementation of mask mandates. In this manner, this study does not draw conclusions regarding changes in search behavior leading up to the mandate.

### Ethical Considerations

This study was not considered human participant research as it used anonymous, publicly available web-based search data and, as such, was exempt from human participant approval.

## Results

### Mask Mandate Implementation and Statewide Demographic Information

**Figure 1:**
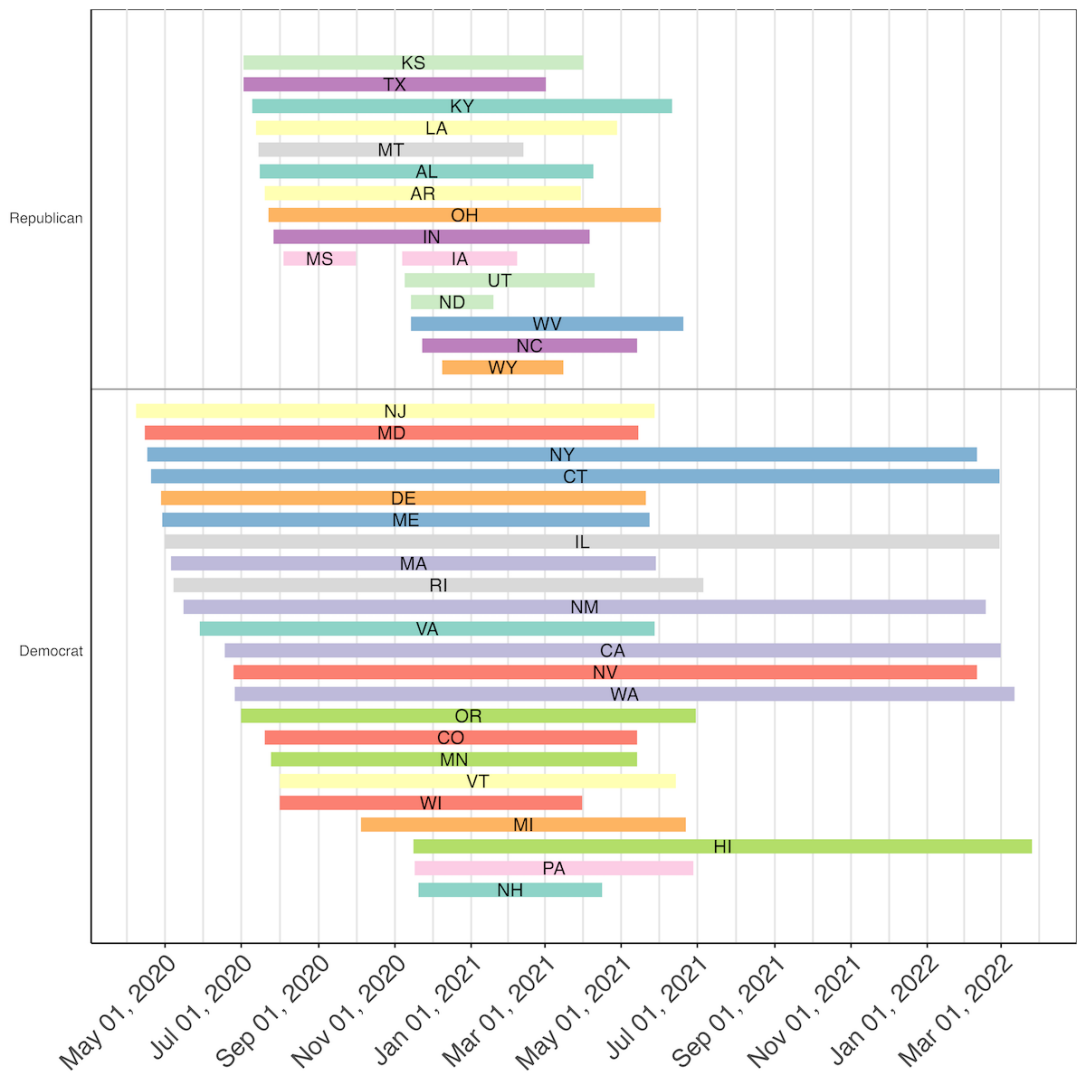
Statewide mask mandate implementation timeline. Colored bars represent the time during which a statewide mask mandate was in effect for a given state, indicated by its abbreviation. Data are reflective of the first statewide mask mandate implemented by each state—some states ended their original mandate and reinstituted it at a later date. AL: Alabama; AR: Arkansas; CA: California; CO: Colorado; CT: Connecticut; DE: Delaware; HI: Hawaii; IA: Iowa; IL: Illinois; IN: Indiana; KS: Kansas; KY: Kentucky; LA: Louisiana; MA: Massachusetts; MD: Maryland; ME: Maine; MI: Michigan; MN: Minnesota; MS: Mississippi; MT: Montana; NC: North Carolina; ND: North Dakota; NH: New Hampshire; NJ: New Jersey; NM: New Mexico; NV: Nevada; NY: New York; OH: Ohio; OR: Oregon; PA: Pennsylvania; RI: Rhode Island; TX: Texas; UT: Utah; VA: Virginia; VT: Vermont; WA: Washington; WI: Wisconsin; WV: West Virginia; WY: Wyoming.

Figure 1 displays a comparative timeline including each of the 39 states that implemented a statewide mask mandate. Fewer Republican states had a mask mandate than Democratic states. Moreover, Republican states that did have a mandate were generally delayed in their implementation, and the mandates were implemented for a shorter period than in Democratic states. Indeed, mask mandates in Republican states were in effect for an average of approximately 206 (SD 89) days in comparison with 430 (SD 162) days in Democratic states. New Jersey was the first state to implement a statewide mask mandate on April 8, 2020. Note that these mask mandates reflect policies that were effective at the state level—some states (eg, Georgia) had mandates at the city or county level, but these mandates were not considered under this investigation. In addition, some states may have ended their initial mask mandate and reinstituted it; however, only the first statewide mask mandate was considered and, therefore, reflected in Figure 1. The exact dates of when each mask mandate was in effect, as well as other state-level information included in these analyses (ie, political party elected, COVID-19 cases and deaths, urbanization, and per capita income), can be found in Table S1 in Multimedia Appendix 2.

### Search Term Preprocessing

The results of the missingness analyses can be found in Table S1 in Multimedia Appendix 1. After preprocessing the raw Google Trends data for the 19 aforementioned mental health search terms, 12 terms (“anxiety,” “depression,” “ocd,” “angry,” “afraid,” “restless,” “irritable,” “tense,” “tired,” “insomnia,” “suicidal,” and “suicide”) had <5% of values missing for aggregated daily hit counts. In other words, throughout all days from March 24, 2020, to March 29, 2021, for each of the 50 states, <5% (19/371) of days did not have a hit count associated with them for these terms. Consequently, data for these terms were included in the modeling portion of the analysis after performing zero imputation, as outlined in the *Outcome Calculation and Imputation* section. All other terms were dropped because of more extensive missingness that may have influenced the model results and comparisons. In addition, at the hourly level, all the 12 included terms had approximately 1% of values missing.

**Figure 2:**
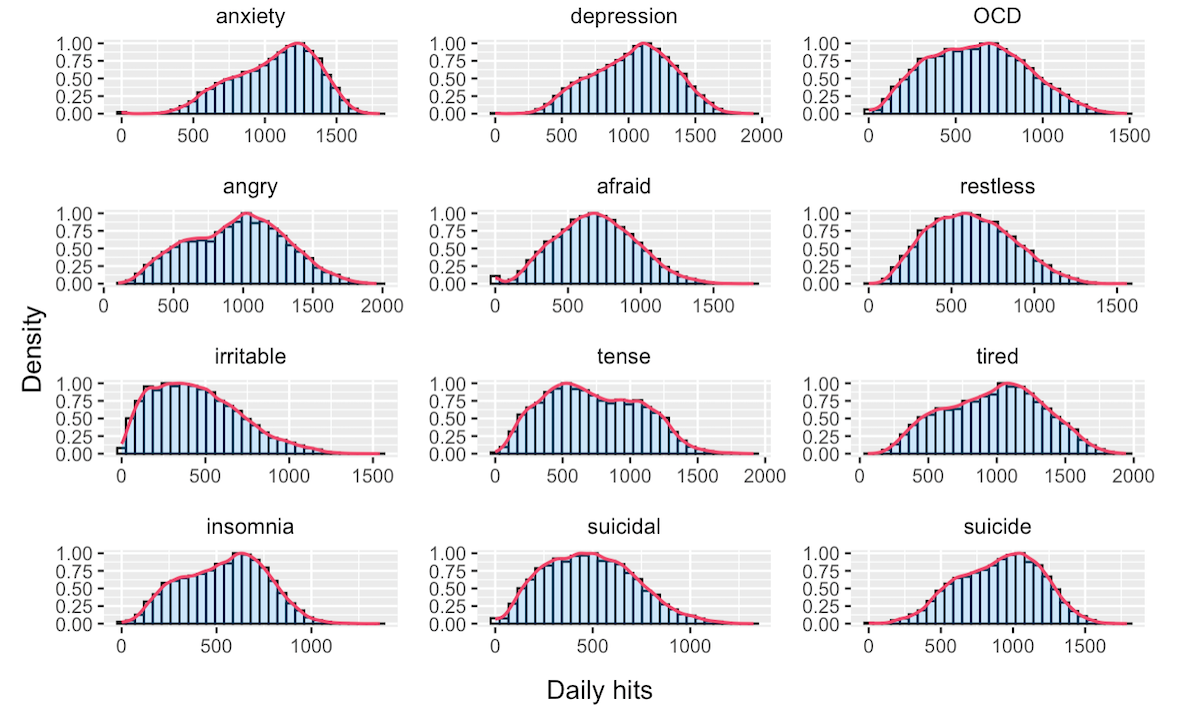
Distributions of daily hit counts for mental health search terms across all states. Each search term–based subplot is a histogram with a density estimate curve superimposed (in red) corresponding to the collection of all daily hit count values across all states for that term. The y-axis is normalized such that the highest value of both the histogram and the density curve is 1. OCD: obsessive-compulsive disorder.

Figure 2 shows the distribution of daily hit counts collapsed across all states for each of the 12 mental health search terms included in the modeling analysis. Distributions of the outcomes varied in central tendency and skewness, but in general, the data were nonnormal and overdispersed, suggesting appropriate use of a negative binomial model fit in GAMM analyses. Figures S1 and S2 in Multimedia Appendix 3 plot the distributions of the daily hit counts collapsed across all states for the physical health terms both related and unrelated to COVID-19 included in modeling.

After preprocessing the raw Google Trends data for the non–COVID-19 physical health terms, 17 terms (“allergic,” “angina,” “apnea,” “bleeding,” “blister,” “bruising,” “constipation,” “discharge,” “fracture,” “hemorrhage,” “incontinence,” “inflammation,” “itching,” “lesions,” “rash,” “spasms,” and “swelling”) had <5% of values missing for daily aggregated hit counts. In addition, after preprocessing the raw Google Trends data for the COVID-19–related physical health terms, 9 terms (“bloating,” “congestion,” “coughing,” “diarrhea,” “dizzy,” “fainting,” “fever,” “strep,” and “stuffy”) had <5% of values missing for daily aggregated hit counts. Accordingly, the 17 non–COVID-19 physical health terms and the 9 COVID-19–related physical health terms were zero-imputed and incorporated into subsequent modeling. Table S2 in Multimedia Appendix 1 displays the full results of the missingness analyses for physical health terms both related and unrelated to COVID-19.

### Mask Mandates, Political Party Affiliation, and Mental Health Search Activity Models

**Figure 3:**
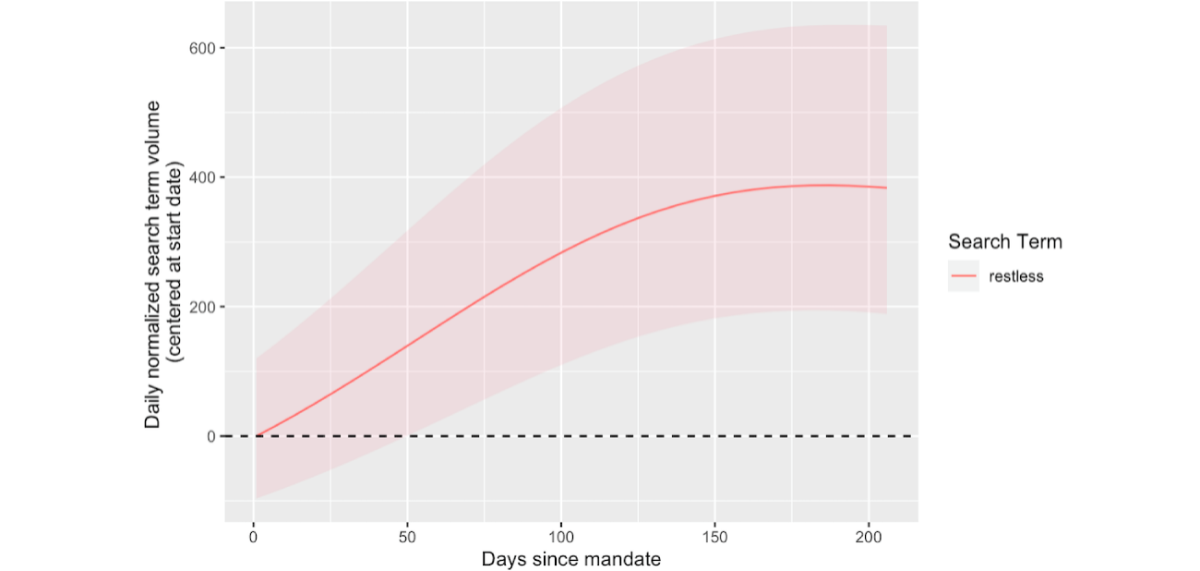
Significant changes in mental health search behavior related to mask mandates. This figure depicts overall changes in state-specific search term behavior relative to time (in days) since mask mandates going into effect. Only search terms with significant changes are plotted. Centering was performed by subtracting the value from day 0 for each term from its respective estimates; thus, changes in search term behavior in the figure are relative to the implementation date of the mask mandate. The 95% CIs are depicted with shading. A horizontal dashed line is drawn at y=0 to depict no change from baseline search volume. The x-axis indicates relative time since the beginning of a mask mandate on a state-by-state basis.

A GAMM model was run, in which the search volume of each of the 12 mental health search terms served as the outcome. The full summary of the model output can be obtained from the authors upon request; however, this analysis focused on the smooth terms that correspond to mask mandate and the interaction of mask mandate with political party elected, as outlined in the *GAMM Approach* section. The results for the smooth term corresponding to mask mandate (s_3_) can be found in Table 1. The search volume for 17% (2/12) of search terms, “anxiety” and “restless,” had significant nonlinear associations with the timing of mask mandates. Analysis of the 95% CI for the estimated change in search volume for “anxiety” revealed that the trend in search volume did not differ significantly from those when the mask mandate was first implemented as the CI contained 0 at all values. However, the trend in search volume for “restless” was found to be significant. As shown in Figure 3, the trend is characterized by a steady increase directly after the mask mandate was implemented, peaking at approximately 400 searches above the initial search volume at approximately 6 months after the mandate and leveling off shortly thereafter.

**Figure 4:**
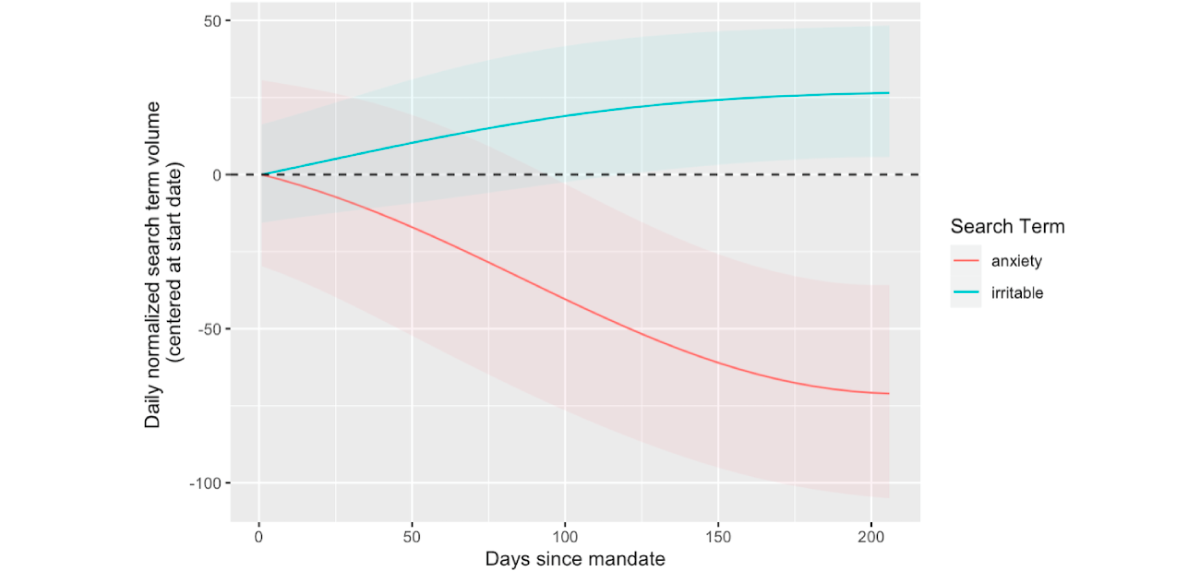
Significant changes in mental health search behavior related to mask mandate and political party interaction. This figure depicts overall changes in state-specific search term behavior relative to time (in days) since mask mandates going into effect. Only search terms with significant changes are plotted. Centering was performed by subtracting the value from day 0 for each term from its respective estimates; thus, changes in search term behavior in the figure are relative to the implementation date of the mask mandate. The 95% CIs are depicted with shading. A horizontal dashed line is drawn at y=0 to depict no change from baseline search volume. The x-axis indicates relative time since the beginning of a mask mandate on a state-by-state basis.

The results for the smooth term corresponding to the interaction of mask mandate and political party affiliation (s_4_) can be found in Table 1. The search volume for 58% (7/12; “afraid,” “anxiety,” “insomnia,” “irritable,” “restless,” “suicide,” and “tense”) search terms had significant nonlinear associations with the timing of mask mandates for Republican states in comparison with Democratic states. Figure 4 plots the associations among these search terms. Of these 7 search terms, 5 (71%; “afraid,” “insomnia,” “restless,” “suicide,” and “tense”) did not differ significantly from baseline (0 contained in the 95% CIs) and, thus, are not plotted in Figure 4. Of the remaining 2 terms, the search volume of “anxiety” decreased significantly after the mask mandate for Republican states relative to Democratic states, dropping to roughly 65 searches below baseline after 6 months. In addition, the search volume of “irritable” significantly increased after the mask mandate for Republican states relative to Democratic states, increasing to roughly 25 searches above baseline after 5 months before leveling off. In sum, although 58% (7/12) of the terms exhibited significant nonlinear associations with the timing of mask mandates for Republican states relative to Democratic states, only “anxiety” and “irritable” exhibited changes in volume that differed significantly from their respective baseline levels.

**Figure 5:**
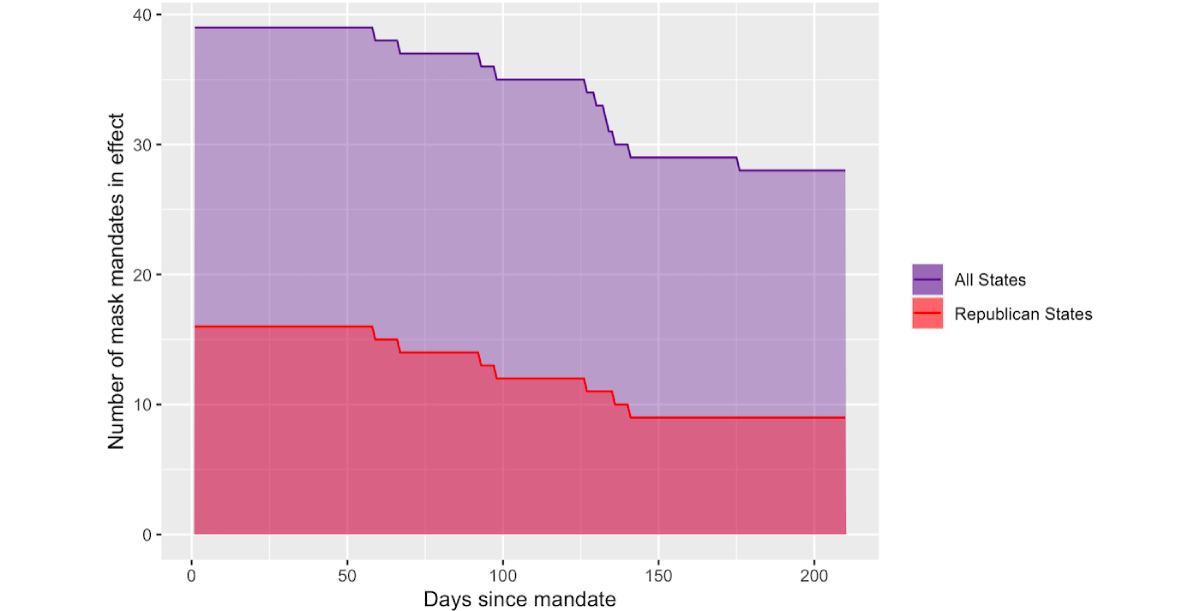
Number of states with mask mandates in effect relative to date of implementation. This figure depicts the number of states with mask mandates in effect after a given length of time since the mandate went into effect, with the purple line and shaded region corresponding to all states (both Republican and Democratic) and the red line and shaded region corresponding to only Republican states. The figure can be interpreted as follows: 39 states had mask mandates (with 16 of these being Republican states), but after 210 days, only 28 of these mandates were still in effect (with 9 of these in Republican states).

Figure 5 displays the number of states with mask mandates relative to the date of mask mandate implementation, or when the mandates first became effective in each state. A total of 39 states had mask mandates initially, with 16 of these states being Republican. After 210 days, or roughly 7 months, 28 of these mandates were still in effect, with 9 of these mandates being in Republican states. In the context of this analysis, as the associations shown in Figures 3 and 4 represent the difference between states with and without mask mandates and between Republican states with mandates and Democratic states with mandates, respectively, there were more states “driving” these associations earlier in time relative to mandate implementation than later in time. It is important to acknowledge the attrition of mandates over time as any significant changes in search volume that happened earlier in time relative to mandate implementation reflect changes that were common across a greater number of states, whereas any significant changes in search volume occurring later in time reflect changes that were common across fewer states. For instance, 210 days following the mandate, only the 9 Republican states with mask mandates were factored into the model that yielded the associations in Figure 4 compared with 16 states at day 0.

Table 1 shows the results of assessing the model fit, in which the marginal *R*^2^ and conditional *R*^2^ were calculated for each search term model. As indicated by the marginal *R*^2^ values, the fixed effects of the models accounted for roughly between 11% and 20% of the variance in search term volume across models. The conditional *R*^2^ values show that the combined fixed and random effects of the models explain roughly between 50% and 76% of the variance in search term volume. These values suggest that (1) the models explain a considerable portion of the variance in mental health search term volume and, (2) although the random effects may explain most of the variance in the outcome for each model, the fixed effects still explain a sizable portion of the variance in the outcome. This latter point provides confidence that the associations between mask mandate and political party elected revealed in Figures 3 and 4 are unlikely to be owing to chance or random noise in the data.

**Table 1:** Results from mental health search term generalized additive mixed models for mask mandate and for mask mandate and political party interaction terms^a^.

Search term and smooth term^b^			EDF^c^		Reference df^d^		*F* statistic		*P* value		Marginal *R*^2^		Conditional *R*^2^
“**anxiety”**													
	Mask mandate	0.005		3		1.181 × 10^12^		.04		0.122		0.565	
	Mask mandate × political party elected	1.917		3		1.234 × 10^17^		.001		—^e^		—	
“**depression”**													
	Mask mandate	2.678		3		5.541 × 10^13^		.47		0.114		0.628	
	Mask mandate × political party elected	1.296		4		7.583 × 10^11^		.17		—		—	
“**ocd”**													
	Mask mandate	0.000		4		0.000		.32		0.160		0.668	
	Mask mandate × political party elected	0.346		4		2.078		.20		—		—	
“**angry”**													
	Mask mandate	2.206		4		8.217 × 10^2^		.21		0.196		0.747	
	Mask mandate × political party elected	0.003		4		0.000		.99		—		—	
“**afraid”**													
	Mask mandate	0.000		4		39.448		.95		0.121		0.498	
	Mask mandate × political party elected	3.021		4		5.374 × 10^13^		<.001		—		—	
“**restless”**													
	Mask mandate	2.897		3		5.487 × 10^13^		<.001		0.107		0.556	
	Mask mandate × political party elected	3.450		4		1.688 × 10^13^		.002		—		—	
“**irritable”**													
	Mask mandate	0.000		4		0.000		.07		0.155		0.677	
	Mask mandate × political party elected	1.589		4		1.008 × 10^3^		.003		—		—	
“**tense”**													
	Mask mandate	0.000		4		0.000		.06		0.120		0.531	
	Mask mandate × political party elected	2.970		4		1.248 × 10^3^		.003		—		—	
“**tired”**													
	Mask mandate	0.001		4		0.000		.32		0.174		0.758	
	Mask mandate × political party elected	1.964		4		224.819		.37		—		—	
“**insomnia”**													
	Mask mandate	2.509		4		3.236 × 10^11^		.06		0.120		0.703	
	Mask mandate × political party elected	2.304		4		1.535 × 10^11^		.02		—		—	
“**suicidal”**													
	Mask mandate	0.001		4		0.000		.32		0.152		0.612	
	Mask mandate × political party elected	0.000		4		0.000		>.99		—		—	
“**suicide”**													
	Mask mandate	0.007		4		0.002		.06		0.116		0.456	
	Mask mandate × political party elected	2.333		4		145.630		.003		—		—	

^a^Numerical values were obtained from the output of the summary() function called on *mgcv bam* model objects in R (R Foundation for Statistical Computing). All *P* values were adjusted to correct for multiple hypothesis testing using the Benjamini-Hochberg method. Any *P* value <.05 is considered to be statistically significant.

^b^Significant values for the “mask mandate” smooth term (s_3_) represent the difference between what would have happened in a state with a mask mandate and what would have happened in the absence of the mandate, and significant values for the “mask mandate × political party elected” smooth term (s_4_) represent the difference between what would have happened in a Republican state with a mask mandate and what would have happened in a Democratic state with a mask mandate.

^c^EDF: estimated df; model-estimated residual df, with 1 corresponding to a linear relationship with the time trend and EDF>1 corresponding to a nonlinear relationship with the time trend.

^d^Equal to the number of model data minus the model df.

^e^The *R*^2^ values are for the model and are not specific to a given smooth term.

### Mask Mandates, Political Party Affiliation, and Physical Health Search Activity Models

Additional GAMMs were fit for physical health search terms to determine whether the associations between mask mandate and political party elected were unique to mental health–related search term activity. For the physical health terms not related to COVID-19, a total of 17 GAMMs were fit—one model with the search volume of each individual term as the outcome. In an identical manner to the mental health search terms, the analysis focused on the smooth terms that corresponded to mask mandate and the interaction of mask mandate with political party elected. The search volume of 24% (4/17) of the search terms—“allergic,” “bruising,” “constipation,” and “rash”—had significant nonlinear associations with the timing of mask mandates. Figure S1 in Multimedia Appendix 4 plots the associations among these search terms. Regarding these 4 terms, the search volume for “allergic” and “rash” did not deviate significantly from baseline at any point (0 in the 95% CI at all time points). However, the search volume for “constipation” increased slowly after the implementation of a mask mandate, peaking approximately 7 months following the mandate. The search volume for “bruising” initially decreased in the 4 months after implementation of a mask mandate, but the volume approached baseline levels in the 4th through 7th months following the mandate, still staying below baseline levels. In addition, the search volume for 12% (2/17) of terms, “constipation” and “fracture,” had significant nonlinear associations with the timing of mask mandates for Republican states in relation to Democratic states, plotted in Figure S1 in Multimedia Appendix 5. The search volume for “fracture” did not deviate significantly from baseline at any point, but the search volume for “constipation” steadily decreased after the implementation of the mask mandate, ultimately becoming significantly different from baseline after 6 months.

For the COVID-19–related physical health terms, 9 GAMMs were fit, with each model using the search volume of 1 of the 9 COVID-19–related physical health search terms as the outcome. The search volume of 22% (2/9) of search terms, “bloating” and “coughing,” had significant nonlinear associations with the timing of mask mandates, plotted in Figure S1 in Multimedia Appendix 6. The search volume for “bloating” decreased steadily after the implementation of the mask mandate, whereas the search volume for “coughing” did not significantly deviate from baseline at any point and, thus, was not plotted. In addition, the search volume of 78% (7/9) of terms—“bloating,” “congestion,” “coughing,” “dizzy,” “fainting,” “fever,” and “strep”—had significant nonlinear associations with the timing of mask mandates for Republican states in relation to Democratic states. However, the search volume for *none* of these terms was significantly different from baseline levels, and thus, no terms were plotted for the associations with the timing of mask mandates for Republican states in relation to Democratic states.

## Discussion

This analysis examined the associations between statewide mask mandates implemented to abate the spread of COVID-19 and changes in web-based mental health search behavior and whether these associations differed across political parties. Google Trends data from March 2020 to March 2021 were used in combination with GAMMs to investigate the associations among mask mandates, political party elected, and changes in mental health search volume. These data provided the framework for an intensive longitudinal analysis that offered quantitative insights into the short- and long-term influences of mask mandates on mental health, as well as an investigation of differences that exist in these associations with political party elected in a state-specific manner. The authors believe that this analysis is the first of its kind to directly examine the associations among mask mandates, political party elected, and changes in mental health activity throughout the COVID-19 pandemic. The results of the GAMMs indicated that searches for “restless” dramatically increased after the implementation of a mask mandate. In addition, Republican states saw an increase in search volume for “irritability” and a decrease in search volume for “anxiety” after statewide mask mandates relative to Democratic states. These associations likely reflect considerably large changes in anxiety-related search behavior in response to mask mandates among the population of the United States. However, most mental health search terms did not exhibit significant changes in search volume in relation to volume before mask mandate implementation (11/12, 92% of the terms) and in relation to volume before mask mandate implementation by political party affiliation (10/12, 83% of the terms).

Although “restless,” “irritability,” and “anxiety” were the only search terms exhibiting significant changes in volume from when mask mandates were implemented, it is important to note that the GAMM results revealed that 2 mental health terms (“anxiety” and “restless”) had significant nonlinear associations with the timing of mask mandate implementation, and 7 terms (“afraid,” “anxiety,” “insomnia,” “irritable,” “restless,” “suicide,” and “tense”) had significant nonlinear associations with the timing of mandate implementation in Republican states relative to Democratic states (Table 1), all after correcting for multiple hypotheses. The main difference with these findings is that only “restless,” “anxiety,” and “irritable” also exhibited *significant nonlinear changes from baseline search volume*, as determined by the 95% CIs (Figures 3 and 4). The fact that many mental health terms exhibited nonlinear associations with the timing of mask mandate implementation is still an interesting finding as this provides insights into how these mental health symptoms fluctuate over time—particularly that these fluctuations are not linear.

The search volume of 12% (3/26) of physical health search terms had significant nonlinear associations with the timing of mask mandates that deviated significantly from baseline, whereas the search volume of only 4% (1/26) of terms had significant nonlinear associations with the timing of mask mandates in Republican states relative to Democratic states that deviated significantly from baseline. Thus, these findings suggest that mask mandate and political party elected had more unique and significant nonlinear associations with mental health search activity than with physical health search activity. In addition, of the 26 total physical health search terms, only 1 (4%), “constipation,” exhibited a significant increase in search volume following mask mandates. Importantly, none of the COVID-19–related physical health terms exhibited increases in volume, suggesting that both mask mandate and political party elected were uniquely associated with greater changes in mental health symptoms. Furthermore, given that the only 3 mental health terms characterized by significant changes in search volume trajectory from baseline (“restless,” “anxiety,” and “irritable”) capture a unified, overarching psychological construct (anxiety), it is likely that these findings are not spurious but rather holistically reflect differences in anxiety-related search behavior following statewide mask mandates. This finding may be informative to leaders and policy makers in the event that pervasive, population-level mandates are ever again necessary as these results suggest that it may be particularly worthwhile to monitor anxiety-related symptoms among the population and provide additional support for these symptoms following mandate implementation. However, given that there were relative decreases in search volume for “anxiety” itself, future research may be needed to investigate the anxiety-related impacts of mask mandates more carefully (eg, at local levels) to better understand the anxiety-related support that should be provided.

Despite increases in anxiety-related search behavior in relation to the timing of statewide mask mandates, there was no significant increase in searches for “anxiety” itself, and in fact, there was a significant decrease in searches for anxiety following mask mandates in Republican states compared with Democratic states. Other studies have found that Republicans were less concerned about catching COVID-19 relative to Democrats as well as less likely to adhere to masking guidelines than Democrats [44,45]. This Republican sentiment could be reflected in decreased searches for “anxiety” relative to Democrats as the pandemic progressed as these findings may indicate that Republicans were relatively less anxious about COVID-19 and masking as a means of preventing its spread. Along with this, considering that mask mandates can be viewed as restrictive [18,19], as well as research that has shown negative sentiment among Republicans toward masking throughout the pandemic [46] and research suggesting greater Republican aversion to the enforcement of mask mandates relative to Democrats [47], this may suggest that Republicans were more likely to feel irritated by mask mandates relative to Democrats, which in turn was reflected in a slight increase in search volume for “irritability” following mandate implementation. In summary, given the exploratory aims of this study, it is interesting to note that the search volume of “anxiety” and “irritability” exhibited differential relationships with mask mandates and political party elected, trends that aligned with existing evidence of political party in relation to masking. The differential changes in “anxiety” and “irritability” could be explained in part by the fact that “irritability” represents more specific emotional states of anger or annoyance within the broader construct of “anxiety,” which itself may reflect a wider array of behavioral responses.

The most notable finding from these results was the significant increase in search volume for “restless” following mask mandate implementation (Figure 3). Searches for “restless” may have increased following a mandate as people were becoming more concerned with others adhering to the new guidelines as well as concerned about the fact that the implementation of a mask mandate may cause others to neglect other health measures such as physical distancing or hand hygiene [48]. It is also possible that people were becoming more restless the longer the mandates were in effect, aligning with research showing increased restlessness as quarantine persisted early in the pandemic [49]. Furthermore, it is possible that there was an increased search volume for “restless” because it was being searched in other contexts. For instance, there is evidence of increased restless leg syndrome—which itself is a nervous condition—because of social distancing measures during the pandemic [50].

Despite the significant changes in anxiety-related mental health searches, it is worth noting that most mental health terms did not exhibit significant changes in search volume from baseline relative to the timing of statewide mask mandates across all states, with 92% (11/12) of terms not having significant changes relative to baseline (Figure 3). Similarly, only 17% (2/12) of mental health terms exhibited significant changes from baseline search volume related to the timing of mask mandates in Republican states relative to Democratic states*,* which nevertheless aligns with other studies that have found differences in the mental response to the pandemic across political parties [51]. Given that most associations were not significant, these analyses do not suggest that there is an overwhelming impact of mask mandates on population-level mental health–related search behavior in the United States. However, when considering why there were fewer significant changes in mental health search volume related to the timing of statewide mask mandates, it is important to note that just because there were no *significant* changes does not mean that mask mandates did not influence mental health search behavior. Owing to the varying time of implementation of statewide mask mandates (Figure 1) as well as other confounding factors not accounted for by these analyses, it is possible that this modeling framework could not decipher state-level associations between mask mandates and mental health searches, but a more fine-grained analysis of within-state search activity may reveal significant associations. Furthermore, these analyses examine associations at the US population level, and despite the efforts to account for covariates across states, there is still likely a large amount of heterogeneity within each state (ie, at the county or community level) in terms of the covariates examined (ie, political party elected, COVID-19 cases and deaths, urbanization, and per capita income). Therefore, the fact that these analyses were able to detect some significant population-level associations between mental health search volume and mask mandates is noteworthy as this likely speaks to the magnitude (ie, number of people experiencing changes in search behavior) of the associations at play. Thus, taking these findings together, policy makers at a statewide and local level should consider the anxiety-related impacts that restrictive orders such as mask mandates can cause, being prepared to offer the appropriate mental assistance when such guidelines are deemed necessary for the greater public good.

This study provided an intensive longitudinal analysis of internet search data over roughly the first year of the pandemic in the United States, being the first known study to provide insights into the short- and long-term associations between mask mandates and mental health. Many different search terms were queried to account for a wide array of mental and physical health constructs. However, only search terms with sufficient completion of data were incorporated into the modeling portion of the analysis, which controlled for a variety of factors, including state-specific time associations and other factors known to influence mental health, such as political party affiliation, COVID-19 cases and deaths, urbanization, and per capita income. The resulting *P* values corresponding to smooth terms were adjusted to account for the multiple hypotheses under consideration, reducing the risk of type-1 error. Furthermore, the marginal and conditional *R*^2^ values for each search term model were reported to show that the fixed effects—namely, the terms corresponding to mask mandates and political party affiliation—explained a considerable portion of the variance in search term volume through time. Finally, physical health search terms were included as a means of comparison with mental health search terms. Taking this methodological approach in summary, this analytical approach was strong in that it accounted for other factors that may have influenced the main results of the mental health models, providing more confidence in these results. Nevertheless, it is impossible to have completely eliminated the possibility of spurious associations, but the several aforementioned measures were taken in an attempt to mitigate this likelihood.

In light of the aforementioned strengths, this study also has limitations. Importantly, all search terms exhibited some level of missingness in that hourly search data were not available for a subset of time points in some states; however, when hit counts were aggregated at the daily level, as long as at least 1 hour was present, the day was considered complete, and the other hours were filled with zeros. Although most days had 24 hours of data available, the assumption that all missing data were owing to low search prevalence (ie, 0 hourly hits) may not have been the case at all times. The search terms extracted from Google Trends also may not have captured all contexts or uses of a word (eg, “anxiety” vs “anxious”) and, thus, may not completely capture public sentiment regarding a mental health construct. In addition, this analysis dichotomously coded political party affiliation based on the results of the 2020 presidential election (ie, “political party elected”). However, this may not have accurately reflected state political representations or true voter sentiment within a state. Future analyses may consider coding political party affiliation on a scale based on the proportion of voters aligning with a given party, but for this analysis, the dichotomous approach led to easier interpretation of the findings. Furthermore, although all mask mandates considered in these analyses were statewide, there was a level of heterogeneity in how mask mandates were implemented at the state level (eg, some were enforced when individuals were in public indoor spaces, whereas others were enforced anytime outside the home [30]). Thus, this heterogeneity may not have been fully captured by considering all statewide mask mandates to be the same in terms of their potential mental impact. In addition, the urbanization data were collected from the 2010 US census and, thus, may not have accurately reflected present-day values. Finally, the modeling approach used did not allow for interpretations of search volume trends preceding the mask mandate. On this note, this study does not have any data available pertaining to the volume of mental health search behavior before the beginning of the pandemic and, thus, cannot account for seasonality and other potentially confounding variables related to time-series data in the analyses.

Given the broad longitudinal scope of these analyses (ie, a year’s worth of search term data), there were increased odds of missingness because of low search prevalence or other data acquisition issues. Accordingly, future work may take interest in examining these data in discretized temporal bins without missingness. In this manner, terms that had to be dropped from this analysis could be included, potentially yielding additional insights into the trajectories of mental health search activity. Furthermore, future work may also consider investigating the associations among mental health, mask mandates, and political party elected using greater levels of resolution in the covariates incorporated into the model in this study (eg, within individual states or communities or subsetting regions by level of COVID-19 cases, urbanization, and per capita income). In addition, given that primarily anxiety-related search terms were revealed to have significant associations within this modeling framework, future work may consider specifically investigating the associations between anxiety-related behaviors and pervasive policies or political parties. Nevertheless, the broad, nationally representative, and longitudinal scope of these analyses provided a sound framework with which to reveal overarching changes in mental health via internet search activity and GAMMs.

## Appendix

Multimedia Appendix 1 Analysis of missingness for mental health and physical health search terms.

Multimedia Appendix 2 State-level information incorporated into generalized additive mixed modeling.

Multimedia Appendix 3 Distributions of daily hit counts for all physical health search terms across all states.

Multimedia Appendix 4 Significant changes in physical health search behavior related to mask mandates.

Multimedia Appendix 5 Significant changes in physical health search behavior related to mask mandate and political party interaction.

Multimedia Appendix 6 Significant changes in COVID-19–related physical health search behavior related to mask mandates.

## Abbreviations

EDF: estimated df
GAMM: generalized additive mixed model
TPRS: thin plate regression spline
