# Supplementary material for: Evaluating the Impact of Mask Mandates and Political Party Affiliation on Mental Health Internet Search Behavior in the United States During the COVID-19 Pandemic: Generalized Additive Mixed Model Framework
Source: J Med Internet Res. 2023 Mar 3;25:e40308. doi: 10.2196/40308 (PMC9994425; doi:10.2196/40308)
Supplement: Multimedia Appendix 1 [file jmir_v25i1e40308_app1.docx]

*Table S1: Analysis of missingness for mental health search terms*

| **Search Term** | Percent of Hourly Data Missing (%) | Percent of Daily Data Missing (%) |
| --- | --- | --- |
| anxiety* | 1.18 | 1.79 |
| depression* | 1.05 | 1.66 |
| ocd* | 1.38 | 1.97 |
| hopeless | 19.44 | 19.17 |
| angry* | 0.99 | 1.59 |
| afraid* | 1.97 | 2.49 |
| apathy | 19.17 | 18.99 |
| worthless | 20.32 | 19.68 |
| worried | 13.25 | 13.34 |
| restless* | 0.98 | 1.62 |
| irritable* | 1.05 | 1.68 |
| tense* | 1.04 | 1.68 |
| scattered | 15.21 | 14.78 |
| tired* | 0.94 | 1.59 |
| avoiding | 8.55 | 8.45 |
| procrastinate | 98.78 | 98.77 |
| insomnia* | 1.14 | 1.79 |
| suicidal* | 1.51 | 2.12 |
| suicide* | 1.05 | 1.69 |

*Note:* There were a total of 8904 hours and 371 days for each of the 50 US states considered in this analysis, which leveraged search term counts spanning March 24, 2020, to March 29, 2021. As daily search term count data were constructed by aggregating hourly counts for a given day, daily data were only considered missing if there were no hourly observations for that day. Search terms with ≤5% daily missingness (indicated with an asterisk) were included in the subsequent modeling analysis.

*Table S2: Analysis of missingness for physical health search terms*

| **Search Term** | Percent of Hourly Data Missing (%) | Percent of Daily Data Missing (%) |
| --- | --- | --- |
| abrasion | 8.00 | 8.26 |
| allergic* | 1.01 | 1.62 |
| angina* | 3.11 | 3.53 |
| apnea* | 1.12 | 1.72 |
| bleeding* | 0.99 | 1.59 |
| blister* | 2.83 | 3.25 |
| bruising* | 3.75 | 4.14 |
| conjunctivitis | 4.92 | 5.26 |
| constipation* | 1.14 | 1.66 |
| discharge* | 6.32 | 4.76 |
| earache | 8.61 | 6.90 |
| flatulence | 19.14 | 17.36 |
| fracture* | 6.33 | 5.00 |
| hemorrhage* | 4.41 | 3.10 |
| incontinence* | 4.60 | 3.25 |
| inflammation* | 4.31 | 3.01 |
| itching* | 4.28 | 2.92 |
| lesions* | 4.33 | 2.98 |
| rash* | 4.34 | 2.98 |
| spasms* | 4.60 | 3.20 |
| swelling* | 4.42 | 3.07 |
| syncope | 7.50 | 6.01 |
| bloating* | 5.29 | 3.87 |
| blurry | 7.18 | 5.50 |
| congestion* | 5.32 | 3.90 |
| cough | 100 | 100 |
| coughing* | 5.87 | 4.38 |
| croup | 13.53 | 11.89 |
| diarrhea* | 5.84 | 4.35 |
| dizzy* | 5.83 | 4.35 |
| fainting* | 6.01 | 4.52 |
| fever* | 5.86 | 4.47 |
| pain | 7.60 | 6.44 |
| sneezing | 7.53 | 6.37 |
| strep* | 5.69 | 4.39 |
| stuffy* | 6.24 | 4.86 |
| vomiting | 7.69 | 6.50 |

*Note:* There were a total of 8904 hours and 371 days for each of the 50 US states considered in this analysis, which leveraged search term counts spanning March 24, 2020, to March 29, 2021. As daily search term count data were constructed by aggregating hourly counts for a given day, daily data were only considered missing if there were no hourly observations for that day. Search terms with ≤5% daily missingness (indicated with an asterisk) were included in the subsequent modeling analysis. The missingness values for physical health symptom search terms both related and unrelated to COVID-19 are given.
