# Supplementary material for: Evaluating the Impact of Mask Mandates and Political Party Affiliation on Mental Health Internet Search Behavior in the United States During the COVID-19 Pandemic: Generalized Additive Mixed Model Framework
Source: J Med Internet Res. 2023 Mar 3;25:e40308. doi: 10.2196/40308 (PMC9994425; doi:10.2196/40308)
Supplement: Multimedia Appendix 2 [file jmir_v25i1e40308_app2.docx]

*Table S1: State level information incorporated into generalized additive mixed modeling*


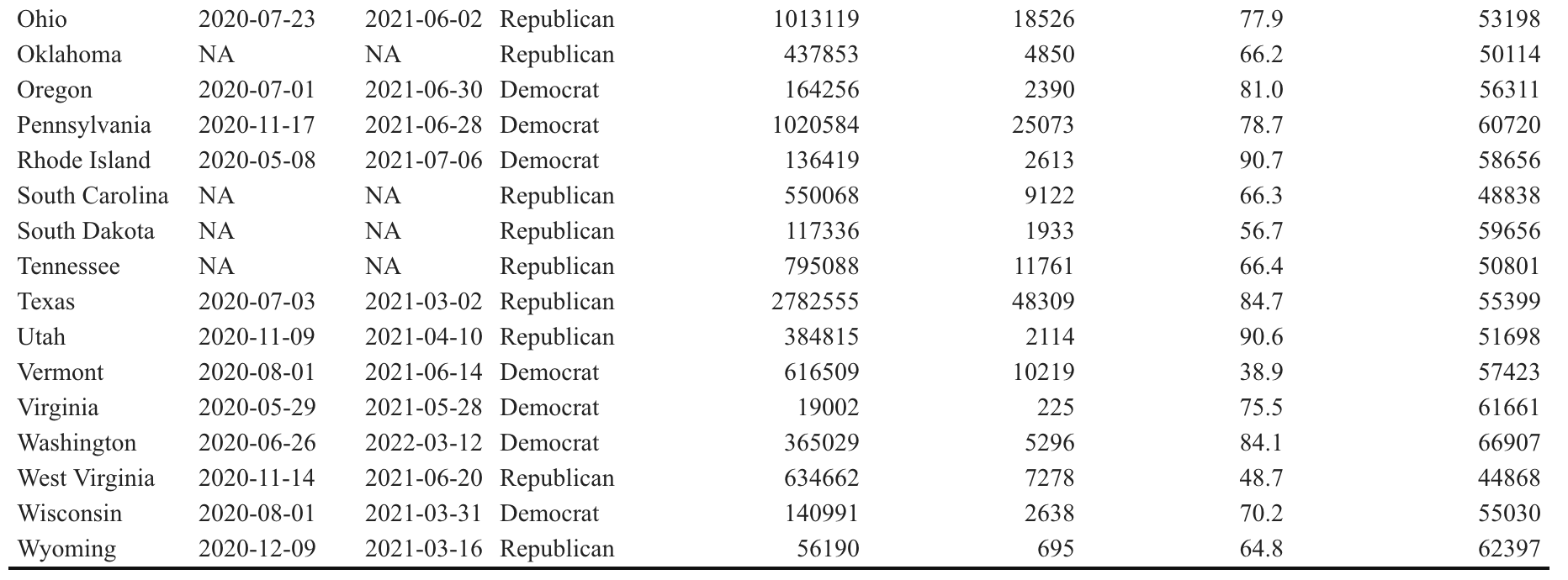

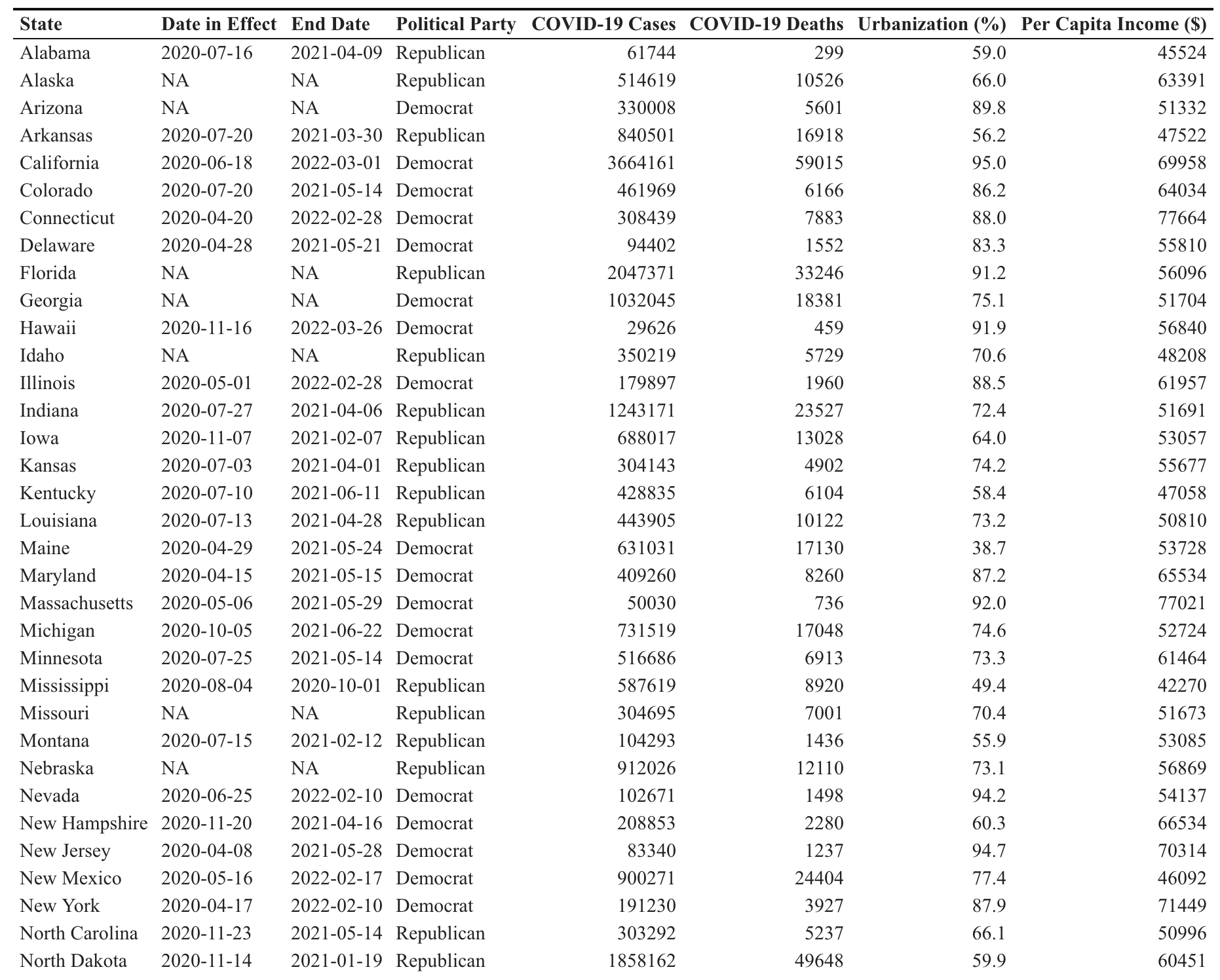
*Note:* States with ‘NA’ for Mask Mandate did not have a statewide mask mandate at any point. COVID-19 Cases and COVID-19 Deaths refer to the cumulative cases and deaths, respectively, in each state as of March 29, 2021—the last date of Google Trends data collection. Political Party is reflective of how each state voted in the 2020 presidential election. Urbanization rates reflect data from the 2010 census, whereas *per capita* income values are reflective of 2020 estimates.
