# Supplementary material for: Evaluating the Impact of Mask Mandates and Political Party Affiliation on Mental Health Internet Search Behavior in the United States During the COVID-19 Pandemic: Generalized Additive Mixed Model Framework
Source: J Med Internet Res. 2023 Mar 3;25:e40308. doi: 10.2196/40308 (PMC9994425; doi:10.2196/40308)

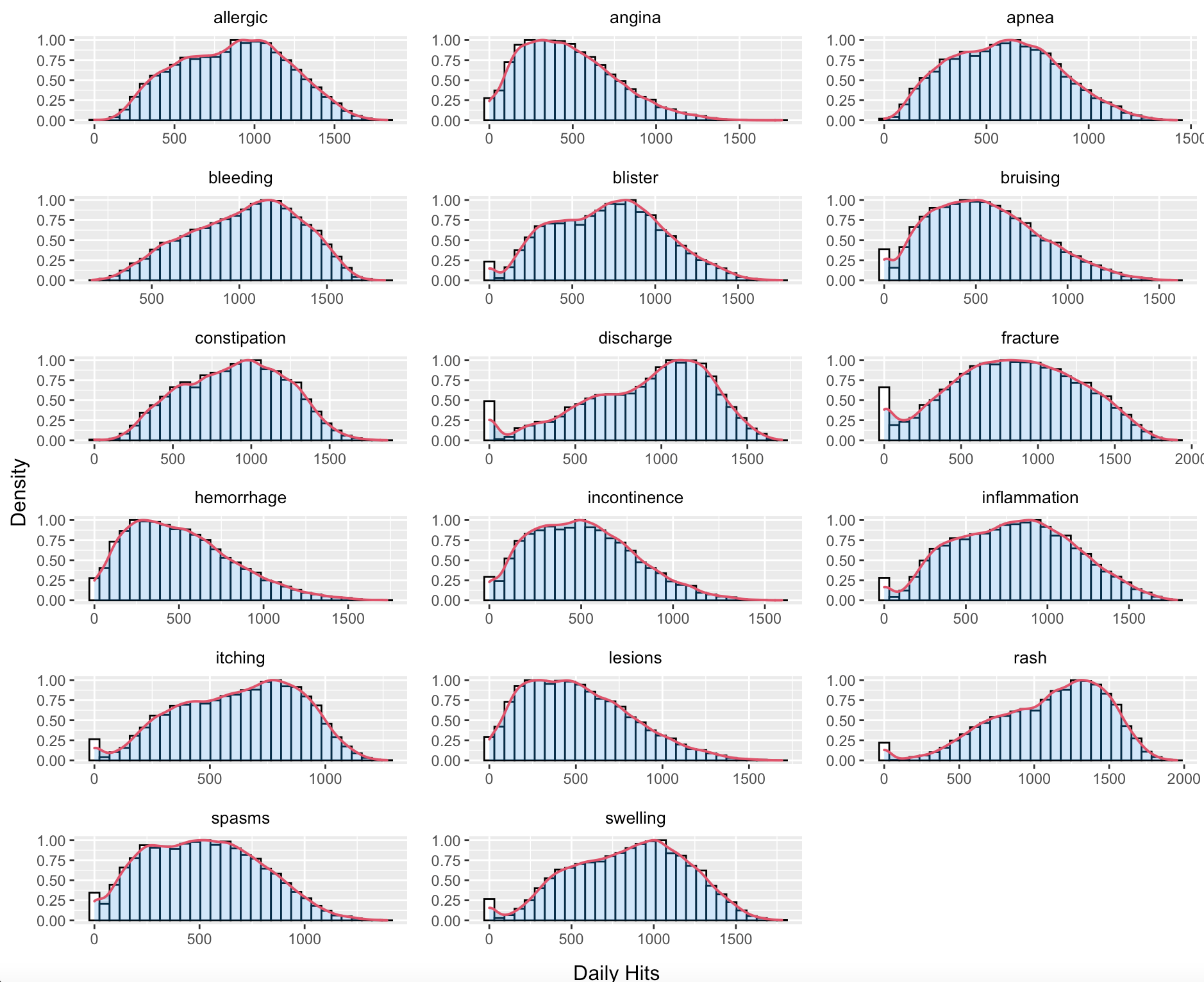
*Figure S1: Distributions of daily hit counts for physical health search terms across all states*

*Note:* Each search term–based subplot is a histogram with a density estimate curve superimposed (in red) corresponding to the collection of all daily hit count values across all states for that term. The y-axis is normalized such that the highest value of both the histogram and the density curve is 1.

*Figure S2:*  *Distributions of daily hit counts for COVID-19–related physical health search terms across all states*

*Note:* Each search term–based subplot is a histogram with a density estimate curve superimposed (in red) corresponding to the collection of all daily hit count values across all states for that term. The y-axis is normalized such that the highest value of both the histogram and the density curve is 1.
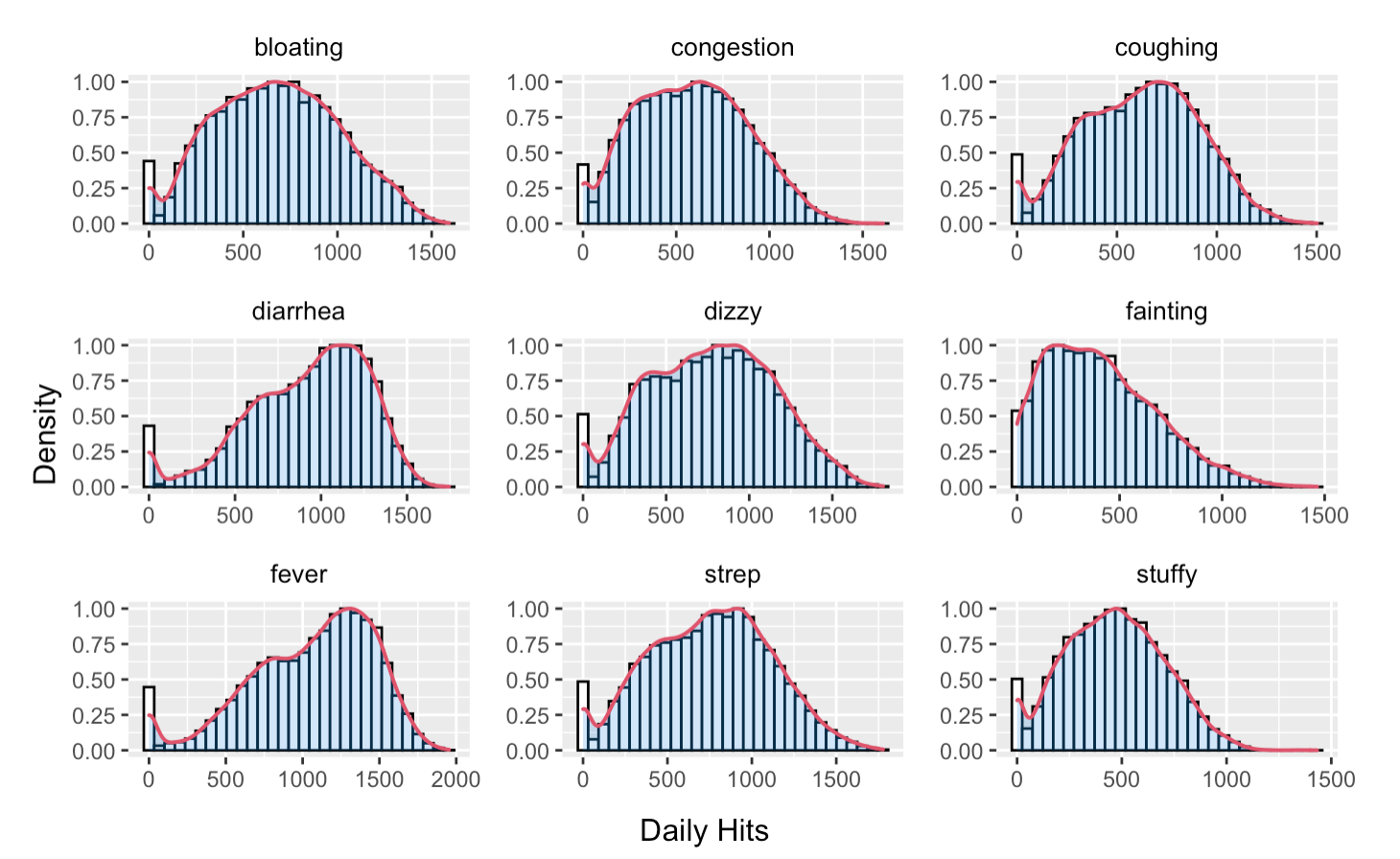

Supplement: Multimedia Appendix 3 [file jmir_v25i1e40308_app3.docx]
