# Supplementary material for: Evaluating the Impact of Mask Mandates and Political Party Affiliation on Mental Health Internet Search Behavior in the United States During the COVID-19 Pandemic: Generalized Additive Mixed Model Framework
Source: J Med Internet Res. 2023 Mar 3;25:e40308. doi: 10.2196/40308 (PMC9994425; doi:10.2196/40308)
Supplement: Multimedia Appendix 4 [file jmir_v25i1e40308_app4.docx]

*Figure S1: Significant changes in physical health search behavior related to mask mandates
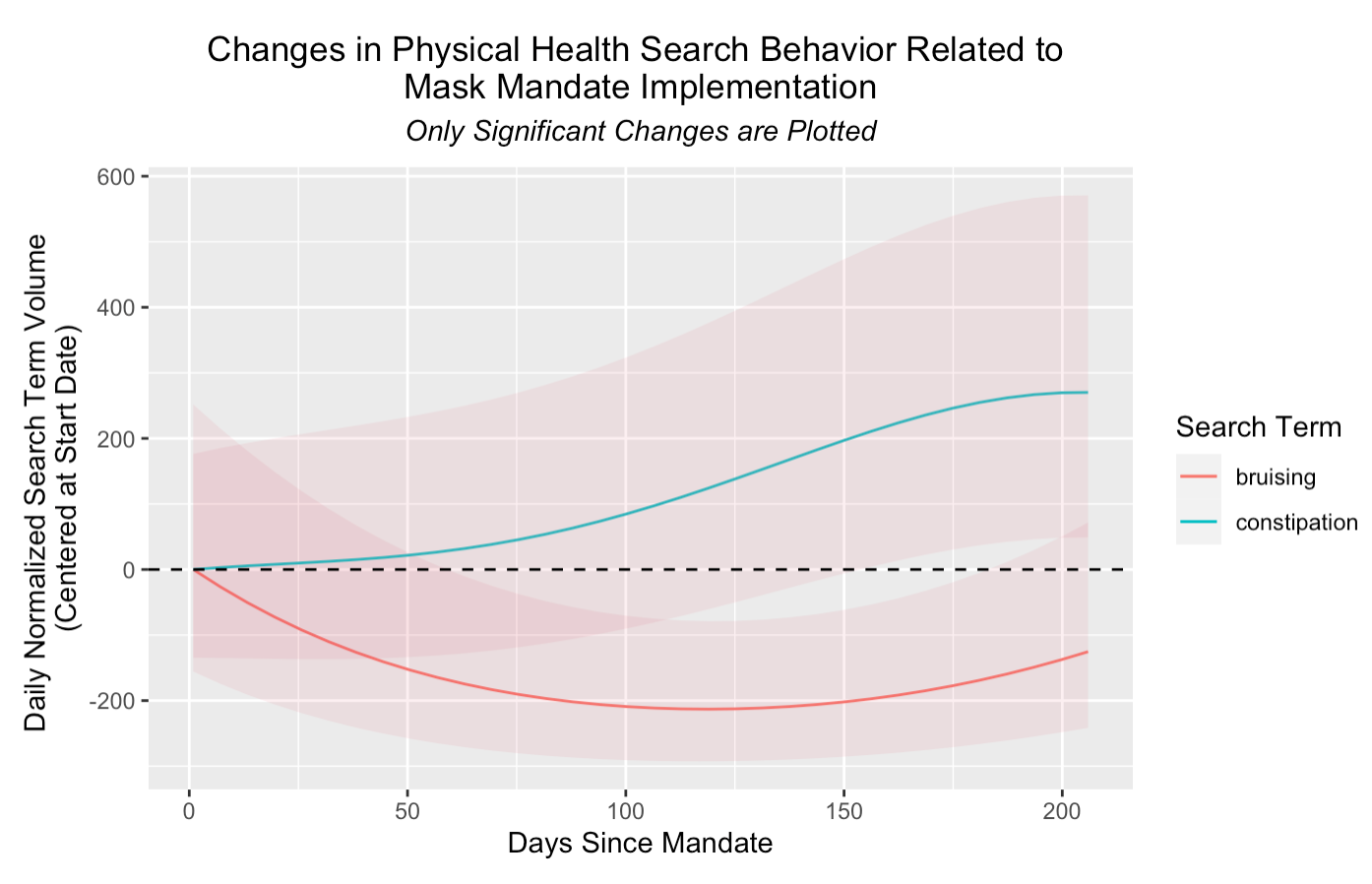
*

*Note:* This figure depicts overall changes in state-specific search term behavior relative to time (in days) since mask mandates going into effect. Only search terms with significant changes are plotted. Centering is performed by subtracting the value from day 0 for each term from its respective estimates; thus, changes in search term behavior in the figure are relative to the implementation date of the mask mandate. The 95% CIs are depicted with shading. A horizontal dashed line is drawn at y=0 to depict no change from baseline search volume. The x-axis indicates the relative time since the beginning of a mask mandate on a state-by-state basis.
